# Review of Economics and Policies of Carbon Dioxide Removal

**DOI:** 10.1007/s40518-025-00252-1

**Published:** 2025-03-07

**Authors:** Soyoung Oh, Jenna Greene, Matthias Honegger, Axel Michaelowa

**Affiliations:** 1 Climate Policy Lab, The Fletcher School, Tufts University, 160 Packard Ave, Medford, MA 02155 USA; 2 Nelson Institute for Environmental Studies, University of Wisconsin-Madison, Madison, WI USA; 3 Perspectives Climate Research gGmbH, Freiburg im Breisgau, Breisgau, Germany; 4 University of Zurich, Zurich, Switzerland

**Keywords:** Carbon dioxide removal, Direct air capture, BECCS, Economics, Integrated assessment models

## Abstract

**Purpose of review:**

Despite the increasing political attention and support, the high costs of many carbon dioxide removal (CDR) technologies remain a barrier to their large-scale deployment. We provide an overview of the economics for two key CDR options – BECCS and DACCS – and review proposed and existing CDR policies to address the “CDR gap” in achieving the long-term temperature goals of the Paris Agreement.

**Summary:**

Although we lack detailed cost breakdowns of actual projects, our review suggests that the cost range for BECCS is generally lower than that for DACCS. The key cost parameter for BECCS is the sustainability of biomass feedstock, and for DACCS the energy intensity.

**Recent Findings:**

Cost estimates for DACCS have increased due to experiences from commercial operation, for BECCS they are increasingly differentiated according to the sustainability of feedstock.

## Introduction

Carbon dioxide removal (CDR) has become increasingly important in climate policy. Recent Intergovernmental Panel on Climate Change (IPCC) reports show that to achieve net zero CO_2_ emissions, the use of negative emissions technologies would be “unavoidable” [1, 2]. Realmonte et al. (2019) similarly noted that reaching 1.5 °C without carbon removal is “infeasible” based on its inter-model assessments [2]. There is a wide range of different CDR options in forest management, industry (e.g., direct air capture with carbon storage, DACCS), and energy, waste, or other biomass processing sectors where a point source can be coupled with carbon capture and storage (e.g., biomass energy with carbon capture and storage, BECCS).

Planting trees tends to be cheaper (ranging between $0 and $240 /t CO_2_ [3], as shown in Table 1) and easier to implement than BECCS or DACCS. However, afforestation and reforestation face a higher risk of reversal than geophysical methods of storage used in BECCS and DACCS [4, 5].

The cost curves of BECCS and DACCS are projected to develop differently over time [6]. Costs of BECCS would initially fall but increase again due to its extensive use of biomass, which is projected to increase in price. Although DACCS starts at a higher level than BECCS, its costs are expected to decrease over time due to scaling effects and efficiency increases. The cost of power is critical, especially for DACCS, which requires high energy inputs, but also in the form of opportunity costs for BECCS given a significant energy penalty associated with the capture process if the CO_2_ capture process configuration is not modified [7].

**Table 1:** Three examples of carbon dioxide removal methods

	Biomass energy with CCS (BECCS)	Direct Air Capture with CCS (DACCS)	Afforestation and reforestation
Approach type	Technology-based	Technology-based	Nature-based
Inherent permanence	High	High	Low-medium
Estimates of carbon removal potential (cumulative to 2100, GtCO_2_)	100-1,170	108-1,000	80–260
CO_2_ capture cost (USD/tCO_2_)	15–400	100–300	0-240
Water requirement	High	Low	High
Land requirement	High	Low	High

Source: Michaelowa et al. (2023) [3] for CO_2_ capture cost (USD/tCO_2_), IEA (2022) [8] for the rest

Due to the high costs and political barriers that we will discuss throughout the paper, CDR has so far only played a limited role in mitigation. This contrasts with integrated assessment model (IAM) scenarios, many of which show a significant role for CDR [2]. We discuss the reasons for this discrepancy below.

First, most IAMs treat CDR technologies as a backstop, meaning that they can be scaled infinitely at a specific price [9, 10]. This is contested by other research [11] that stresses growing negative environmental effects when upscaling, particularly in the case of BECCS [12]. Second, Hollnaicher (2022) criticizes an overly high discount rate in the IAMs, which leads to an overestimation of CDR [13]. Third, the extent and originating sectors of so-called “residual emissions” that are offset by CDR when countries reach net zero differ significantly in modeled scenario pathways [14–19]. Often residual emission estimates are not explicitly defined in long-term national climate strategies, and for those countries that do define them, the level of residual emissions varies widely [16, 17]. Ambiguous amounts of residual emissions and the sectors in which these can take place lead to questions about responsible sectors or entities to deploy or pay for CDR [19]. Importantly, the emissions that are defined as residual come from notions of what is necessary and what is possible in the future, both of which are assumptions with political and justice implications [18]. In considering the amount of CDR needed at the time of net zero, it is important not only to evaluate what are considered residual emissions but also the reasoning behind why these emissions are hard to abate.

Fourth, there is imperfect information. Modelers heavily rely on information provided by industry insiders and lack data points from independent sources. For instance, in Germany, Bochers et al. (2024) noted that the lack of information on market costs for CDR options in the German context posed challenges in accurately estimating marginal removal costs [20]. Further, cost projections for DACCS have long relied on very few original data points [21].

Lastly, the life-cycle environmental impacts of CDR are highly important. Through a review of Life Cycle Assessments (LCAs) of a wide range of CDR options (e.g., afforestation and reforestation, BECCS, DACCS, enhanced weathering, ocean fertilization), Terlouw et al. (2021) highlighted that each technology faces different types of uncertainties [22]. For instance, biomass-related CDR should consider temporal aspects of emissions, while DACCS faces uncertainties regarding the level and origin of energy consumption [22].

Lamb et al. (2024) identify a ‘CDR gap’, assessing policies supporting CDR and finding that these are not sufficient to generate the CDR capacity to limit warming to 1.5 °C [23]. The authors further discussed the competition between political interests aiming at expanding land-based removals and those that want to scale novel methods such as BECCS, DACCS, and biochar.

In this paper, we examine the economic dimension of the ‘CDR gap’ by providing a comprehensive review of the literature on how the understanding of the economics of CDR has evolved and what policy measures have been employed to address the challenge. First, we provide a review of theoretical and empirical cost estimates and projections for BECCS and DACCS. We chose BECCS and DACCS because they are two technologies that are deemed to have reached relatively high technology readiness levels (TRL) (e.g., BECCS: 5–6, DACCS: 6 out of 10) with high inherent storage permanence [1]. Recently, Climeworks has completed two commercial DACCS plants in Iceland (Orca, Mammoth) and aims to further upscale its technology in the United States (Project Cypress in Louisiana, Prairie Compass DAC Hub in North Dakota, and California DAC Hub in California). BECCS projects in operation include Stockholm Exergi in Sweden [24] and Decatur (an ethanol plant with CCS) in the United States [25]. Subsequently, we provide an overview of CDR policies.

## Economics

### Economics of DACCS

The economics of DACCS varies depending on capture technology, energy costs (the price of heat and electricity), location, and whether the captured CO_2_ is stored or used. In this section, we specifically look at the capture phase of DAC; we cover the transport and storage phases in the following section. Theoretical estimates use learning curves. Due to different estimation approaches (e.g., solid sorbent DAC vs. liquid sorbent DAC), transport, storage types (e.g., mineralization vs. Injection into depleted oil and gas reservoirs), source of energy, geographies, labor costs (i.e., affecting construction and capital costs) and timelines the expected range of cost of DACCS in 2030 is wide at $120 to $600/tCO_2_. Further, the limitations of IAMs pose challenges to precisely identifying the DACCS cost. For instance, studies on IAMs make different assumptions on carbon price levels, as well as key factors influencing DACCS costs (energy prices, and heat requirements), while often failing to endogenize innovation dynamics and include the time needed to build plants and drive down the cost through scaling (See Table 2).

**Table 2:** Review of cost estimates of DACCS

Authors	Technologies	Estimated range (cost/ t CO_2_)
Peer reviewed literature		
Sievert et al. (2024) [26]	L-DAC, S-DAC	L-DAC: $226-$544 S-DAC: $230-$835 (at 1 Gt-CO_2_/year cumulative)
J. Young et al. (2023) [27]	S-DAC, KOH BPMED, KKOH-Ca looping, MgO ambient weathering	$1300-$3100 (FOKA) $100-$600 by 2050
R. Young et al. (2022) [28] ^1^	Not specified	$120-$300 by 2030 $90 by 2050
Lackner & Azarabadi (2021) [29]	Not specified	$500-$600 (2019; based on Climeworks) Below $100 by 2040
Sabatino et al. (2021) [30]	L-DAC, S-DAC	$100-$200/ at a large scale
DACCS technology providers		
Climeworks (2024) [31]	S-DAC	Aims to reach $400–600^2^ by 2030 and $250–350 by 2040
Climeworks (2019) [32]	S-DAC	$500-$600 in 2019, and aims to reach $100-$150 in 2030
Carbon Engineering (2018) [33]	L-DAC	$94–232/ at a large scale (non-specified date)

^1^This is a preprint article, it offers immediate access but has not been peer reviewed

^2^Total net removal cost. Climeworks aims to achieve costs of $250–350 per ton of CO_2_ by 2030

Previous learning curve literature has shown that attaining cost reductions of DAC is theoretically possible, as we have seen in other clean energy technologies [34]. The cost of DAC could follow a path similar to other successful technologies if sufficient capital investment could bring down its cost through learning-by-doing [29]. Compared to solar PV modules in the early 1960s, which needed to reduce their cost by more than 100-fold to be competitive with conventional energy sources, the authors note that DAC might be better positioned to lower the costs from $500–600/t CO_2_ as it only needs to come down by 10-fold [29]. While Lackner & Azarabadi (2021) assumed a consistent learning rate for the entire DAC system [29], Young et al. (2022) provide a more disaggregated analysis of the DAC cost by dividing the DAC’s overall cost into initial cost and operation and maintenance costs [28].

Overall, the theoretical estimates from the studies suggest that the cost of DACCS declines over time but not necessarily rapidly enough to reach the targets to limit global warming to 1.5ºC and 2ºC by 2100. Recent literature in the past 1–2 years is also more pessimistic than the previous research [26, 30]. Climeworks (2024), which now has considerable operational experience, estimates no significant cost decrease until 2030 [31]. Young et al. (2023) note that more modular technologies, like modular S-DAC, often have higher learning rates, but this effect is counteracted by lower economies of scale [27]. The authors also showed that cost reductions depend on whether the energy powering DACCS comes from nuclear power or renewables. For instance, Sievert et al. (2024) consider that electricity from solar PV for a liquid solvent DAC plant would contribute to 81-88% of the total net removed cost based on a multi-component one-factor experience curve model [26].

Further, the limitations of IAMs pose challenges to precisely identifying the DACCS cost level needed. For instance, studies on IAMs make different assumptions on carbon price levels, as well as key factors influencing DACCS costs (such as energy prices and heat requirements), while often failing to represent realistic innovation dynamics and the time needed to build plants and drive down the cost through scaling.

The main challenges to lowering the cost of DAC deployment are identified as (1) energy demand and (2) limited markets for CO_2_. Due to the high and essentially unavoidable energy demand of solvent regeneration [30], efficient air contact with the solvent is extremely important. Fasihi et al. (2019) also show that to achieve low DACCS cost, several conditions (e.g., low-temperature heat requirements, declining capital costs, and declining energy requirements) have to be met [35]. Second, as the primary product of DAC is CO_2_, which has limited market demand (i.e., mostly coming from enhanced oil recovery which is unsustainable in the long run due to its negative climate consequences), operating DAC facilities are unlikely to generate sufficient revenues without the creation of new markets and policy support. As a pure climate change mitigation technology DACCS does not provide significant co-benefits. It needs policy instruments that provide revenues either through grants or the acquisition of emissions credits [36].

### Economics of BECCS

BECCS was seen as the only potential “backstop” technology in IAMs until very recently when DACCS appeared in the models, albeit with optimistic cost assumptions. However, BECCS faces challenges of energy conversion losses and limited feedstocks [37].

The cost of BECCS is highly dependent on the geography and the technology, as well as biomass type and energy use. Theoretical estimates using various approaches (e.g., IAM review and process economics) show the per ton cost range of BECCS between $13 and $288 [38, 39]. Fajardy et al. (2021) show that BECCS could play an important role in contributing to emissions reductions, acting as a true backstop technology when carbon prices are near $240/tCO_2_ (See Table 3).

**Table 3:** Review of cost estimates of BECCS

Authors	Technologies	Estimated range (cost/ tCO_2_)
Peer reviewed literature		
IEA (2020)^1^ [39]	Power generation	$56-$64
	Other fuel combustion	$13-$25
	Industry	$79–85
Fuss et al. (2018) [38]	Combustion	$88-$288
	Ethanol	$20-$175
	Pulp and paper mills	$20-$70
	Biomass gasification	$30-$76
Technology providers		
McKaskle & Whittaker (2021) [40]	Ethanol plant (capture using Alstom’s amine process)	Decatur: $18- $24
Ricardo EE & BEIS (2020) [41]	Wood as feedstock for power (post combustion amine)	Drax: about $43 - $66 (£149/MWh-£230/MWh)

^1^IEA compiled these values based on EASAC (2018), Fuss et al. (2018), Haszeldine et al. (2018), Keith et al. (2018), Realmonte et al. (2019)

Compared to DACCS, BECCS has been demonstrated for a relatively longer period at a commercial scale and has reached a lower cost range. For instance, the Illinois Industrial Carbon Capture and Storage project started in 2016 at a low cost due to its proximity to the Mt. Auburn injection site [40]. However, previous literature notes that even though BECCS costs start at a lower level, it is expected to decrease more slowly than DACCS due to the limited availability of feedstocks [6, 38, 42]. If the deployment is only driven by cost considerations, it may pose challenges in sustainability as an increase in BECCS deployment implies the increased use of productive land for bioenergy production. Increased biomass demand may result in unsustainable harvesting practices and displacement effects [43, 44]. Fajardy et al. (2019), however, noted that the indirect land use change is difficult to evaluate without uncertainties as it is highly contextual (e.g., economic conditions, time horizon).

Arguably, BECCS can replace natural gas peaking plants, providing an alternative for balancing renewable energy intermittency issues if there are high carbon prices. However, CDR efficiency can be significantly diminished or even result in net emissions generated in case of significant “carbon debt” caused by land use changes and unsustainable harvesting. Therefore, BECCS is increasingly explored in the context of waste incineration, where no additional biomass demand would be created [45].

### Combined Assessments

In the case of the European Union, Galán-Martin et al. (2020) suggest a mix of implementing BECCS first and deploying DACCS with later starting years for the cost-optimal [46] and maximum removal roadmap as BECCS faces technical challenges to be scaled up [2, 37, 46, 47].

## Policy

### National Plans for CDR

The rise of net-zero targets, set by countries, sub-national governments, businesses, and non-governmental organizations, includes both rapid emissions reductions and implies CDR. However, it is often unclear what share of these targets will be achieved through emissions reduction and how much will come from CDR – in addition to when those removals will happen, through what technologies, and how durable storage will be ensured. Whether CDR targets and emissions reduction targets should be separated to avoid the fungibility of emission reduction and removal credits is disputed [48, 49], but there is agreement that CDR plans need more clarity. Details on both the timing, management of CDR (ibid.), and specificity of targets (including on a sectoral level) are important to build accountability [50].

Governments are increasingly including CDR in their communications to the UNFCCC, including Long-Term Low Emission Development Strategies (LT-LEDS) and Nationally Determined Contributions. In these documents, land-based CDR approaches through the enhancement of natural carbon sinks on land are most commonly mentioned by parties [15, 23, 51, 52]. They often lack detail as to the role of CDR and the policies foreseen to mobilize them [51]. Only 12 countries’ LT-LEDS included an explicit quantification of the envisaged CDR [52]. The levels of CDR included in national plans are significantly lower than the CDR levels in most IAM scenarios that limit warming to 1.5 °C, thus leading to a ‘CDR gap’ [23, 53] as discussed above. Closing this gap will require not only raising the ambition of CDR targets but also the implementation of sufficiently ambitious policies that ensure the necessary conditions for CDR implementation.

### CDR Policy Landscape

CDR technologies vary in terms of technological readiness, economic factors, potential side effects and benefits, and the political and social landscape surrounding them. While many are novel, with low technological readiness levels and active research and development being done, others, such as afforestation and reforestation, have been implemented for centuries and have reached gigatonne scale. Just as the technologies are varied, so too are the policy considerations. The technologies also differ in terms of their political acceptance among the public [54, 55] and policymakers [56], so differentiation in both policymaking and public engagement may be important for the political feasibility of scale-up. Many researchers have therefore emphasized the need to differentiate the strategies and policies for different CDR technologies in the near term to account for these differences [57–59].

For novel CDR approaches, both the technology and the policymaking are in their formative phases [60, 61]. Most existing policy mechanisms and regulations are focused on conventional CDR on land (afforestation, reforestation, and restoration of natural carbon sinks) [62–64] and have a long history, reaching back to well before the start of international climate policy. Although CDR is being considered at a larger scale, with more urgency, and through a larger suite of technologies than past notions of carbon sequestration, past experiences with forestry policies should be considered to learn from past successes and avoid repetition of failures [65].

Market-based approaches involve either emissions trading or baseline and credit mechanisms [3, 64]. In the past, carbon markets were focused on reducing or avoiding emissions, but the Clean Development Mechanism (CDM), introduced in the Kyoto Protocol, included afforestation and reforestation, and in 2011, detailed rules for geological storage under the CDM were agreed upon. In recent years, the explicit inclusion of CDR in carbon markets has been discussed at the international and national level, most prominently in the European Union, which is considering reforming its Emissions Trading Scheme to include CDR through a Carbon Removal Certification Framework. There is an animated debate about which CDR options should qualify. Rickels et al. (2022) propose a Carbon Central Bank serving as a clearing house to supply CDR credits [66]. Another group of authors argues that only novel CDR should be incorporated while conventional land-based CDR should be included in an expanded portfolio of LULUCF regulation [67]. Differentiating between short-term removal activities and durable carbon removal in the Carbon Removal Certification Framework is seen as critical [68].

Voluntary carbon markets have to date been the most successful policy instrument to harness CDR, mostly for forestry, biochar, and DACCS. Prices for DACCS credits have reached over $1,000, and BECCS credits have been trading at several hundred dollars [3].

Public procurement of CDR by governments, ideally through reverse auctions in which CDR project developers submit bids and the developer with the lowest bid price receives the incentive funding in the form of a Contract for Difference, has been proposed in the literature to enable CDR [69]. It is already implemented by Denmark, where the utility company Ørsted won the first BECCS auction, the United States Department of Energy [70], Sweden [71], and Germany [64, 72].

Fiscal incentives for CDR, such as tax credits and subsidies, are primarily focused on conventional, land-based CDR [64] but public funding for CDR demonstration projects has increased in 2024 in some countries, mainly focused on novel CDR methods. The largest of these programs is the DAC Hubs program, a multi-billion dollar grant program through the US Department of Energy [73].

Private and public programs for baseline and credit systems protect against fraudulent activity and ensure that the credits being claimed are real and additional. This is done through the approval of baseline and monitoring methodologies as well as independent validation of project documentation and verification of monitoring reports. For CDR, the key task of such programs is to ensure carbon is being removed and stored durably [3]. Recently, many CDR-specific programs have sprung up (e.g. PuroEarth and Isometric). A survey of CDR programs found that more baseline and monitoring methodologies are available for conventional, nature-based CDR than for novel CDR and that the quality of methodologies differs in both voluntary and compliance markets [74]. Accounting for carbon removal should differ between fossil-based emissions and land-based emissions because of the timescales involved and the permanence of removals [75]. Clarity in programs, their methodologies and independent oversight is critical to ensure the quality of removal credits [74].

### Considerations for CDR Policy

Beyond techno-economic factors, social and political considerations are also critical in designing policies to support CDR, both in terms of which CDR methods that policies focus on and how they will be scaled up [50, 76, 77]. The policy instruments used to support a CDR technology may even impact the public perception and level of support for that technology [78]. Currently, direct subsidies are the preferred policies while integration in carbon markets is proceeding more slowly than expected.

CDR policy will overlap with other policy objectives, such as health, energy security, food security, and environmental sustainability [63]. Consideration of these other goals and focusing on the co-benefits of CDR can shape the future of CDR policymaking and scale-up. Public programs to fund CDR should consider equity and fairness in the funding mechanism to ensure that funding CDR does not exacerbate existing inequality [79]. Because most CDR technologies involve many sectors and scales, incentivizing different technologies based on the co-benefits that come from each method may improve social acceptance [36].

## Conclusion

As policies increasingly support CDR via grants, carbon markets, and regulatory schemes, a favorable environment for crowding in private investments for CDR projects is emerging. Voluntary carbon markets and offtake agreements have been critical to financing the first commercial DACCS plants. Grants for large DACCS projects such as the United States Regional Direct Air Capture Hub will push significant upscaling of DACCS.

The first experiences with the operation of DACCS and BECCS plants have shown an increase in costs compared to previous estimates. This shows that the learning effect focused upon theoretical assessments is only likely to kick in as significant experience with commercial plants is being made. Moreover, the current focus on grants and subsidies may not be sufficient to lead to a rapid cost decrease. Therefore, governments need to ensure that carbon markets play a key role in incentivizing continuous cost reductions.

## Data Availability

No datasets were generated or analysed during the current study.
